# Design and interpretation of eQTL-GWAS colocalisation studies: Lessons from a large-scale evaluation

**DOI:** 10.1371/journal.pgen.1012141

**Published:** 2026-05-08

**Authors:** Guillermo Reales, Jeffrey M. Pullin, Ichcha Manipur, Elena Vigorito, Chris Wallace

**Affiliations:** 1 Cambridge Institute of Therapeutic Immunology & Infectious Disease (CITIID), Jeffrey Cheah Biomedical Centre, Cambridge Biomedical Campus, University of Cambridge, Cambridge, United Kingdom; 2 MRC Biostatistics Unit, University of Cambridge, Cambridge, United Kingdom; University of Miami Miller School of Medicine, UNITED STATES OF AMERICA

## Abstract

Colocalisation analysis integrates GWAS and molecular QTL datasets to identify candidate effector genes. Even with a wide range of molecular QTLs, 40% or more of GWAS loci remain unexplained, leaving a “colocalisation gap”. We systematically characterised two large-scale eQTL colocalisation studies, to describe the determinants of this gap and ultimately inform the selection and design of eQTL studies to close the gap. We analyse over 1.3 million colocalisation tests from Open Targets Genetics (OTG) and perform and analyse colocalisations from 14 immune-mediated disease (IMD) GWAS and 12 diverse immune cell eQTL studies, selected to cover a range of cellular granularities and sample sizes. We find that 50% of GWAS peaks in OTG and 34% in IMDs colocalised and were more likely to colocalise if they were located nearer to genes and had a more common lead variant. Colocalisation was also more likely to occur in disease relevant tissues. The lowest granularity immune cell eQTL studies had the largest sample sizes, the greatest eQTL discovery and produced the largest number of colocalisations, particularly for lower-frequency variants. However, while higher resolution eQTL studies detected fewer eQTLs, each of those eQTLs was more likely to colocalise with a GWAS peak, emphasising the importance of cell specific eQTLs. Indeed, over 50% of colocalisations were found in only one cell type. Overall, our results suggest that a diverse set of cells in different contexts, and large, high granularity studies will be needed to identify remaining colocalisations. In addition, we observed that 47% of GWAS peaks colocalised with multiple genes in OTG and 37% in IMDs. Through simulations, sensitivity analyses, and integration of enhancer-promoter capture data we find that multiple colocalisations likely represent coregulation. While disentangling causality from horizontal pleiotropy will ultimately require experimental perturbation, triangulation using different sources of observational data is likely to be necessary for gene prioritisation.

## Author summary

Most of the genetic variants associated with complex traits are located outside genes, limiting functional interpretation. Genetic colocalisation helps identify candidate causal genes by testing whether the same variant influences a complex trait and the expression of a nearby gene (eQTL). By comparing eQTL studies with different cell-type resolution and sample size, this work shows how study design affects colocalisation results. Overall, 34–50% of complex trait associations showed evidence of colocalisation with at least one gene, with higher rates for variants closer to genes and with more common lead alleles. Over half of detected colocalisations were cell-type-specific. Fine-grained, cell-type–specific datasets capture more specific colocalisations per eQTL, but have reduced power due to smaller sample sizes, whereas low granularity, higher-powered datasets detect more total colocalisations, particularly for rarer variants. This indicates that future progress requires both high resolution and substantial sample size. Among colocalising loci, 37–47% matched multiple genes, implying that these genes may share regulatory mechanisms, or that we were detecting false colocalisations. However, stringent sensitivity analyses suggested false colocalisations were unlikely to explain the majority of multiple gene colocalisations, and independent data supported shared regulation. In these cases, distinguishing causal genes will require experimental validation, but integrating complementary observational datasets can strengthen gene prioritisation.

## Introduction

Genome-wide association studies (GWAS) have identified hundreds of thousands of associations between genetic loci and complex traits. However, mapping the path from genotype to phenotype remains challenging. Over 90% of GWAS variants lie in non-coding regions of the genome, and so cannot be trivially linked to an effector gene and molecular mechanism. Converging evidence suggests that most non-coding GWAS loci act by regulating the expression of nearby genes. A common approach to elucidate the regulatory mechanisms is to integrate summary data from GWAS and expression QTLs (eQTLs) using colocalisation to nominate the likely causal genes for each GWAS signal. Colocalisation aims to determine whether the GWAS trait and eQTL share the same causal variant in a region, with the implicit conclusion that a colocalising gene is likely to lie on the causal pathway for disease.

Despite extensive efforts to link eQTLs from a wide range of tissue types to diverse GWAS traits, most GWAS loci remain unexplained by currently catalogued eQTLs. For example, analyses using GTEx expression data on 49 tissues and covering 5,385 GWAS loci, estimated the proportion of colocalising loci with at least one gene to be 43% [1] or 40% [2], with a median of ~20% loci per trait. Another analysis found that only 25% of autoimmune GWAS loci colocalise with eQTLs in three major immune cell-types from GEUVADIS [3]. These low rates of colocalisation have led to the notion of the so-called “colocalisation gap”: the fraction of GWAS signals with gene regulation potential that cannot be linked to a target gene by colocalisation [4].

Several hypotheses have been proposed to account for the missing colocalisation. One possibility is that eQTL studies have been too small to saturate the eQTL map, leaving colocalisation underpowered, particularly for lower frequency variants [5]. Additionally, we know that different sources for the eQTL study vary in how informative they are for any given GWAS. For example, eQTLs from patient samples are better able to explain disease associated GWAS signals than from healthy control samples [6,7], just as samples from the disease site are more informative than samples of the same cell type from elsewhere in the body (e.g., immune cells from the colon rather than peripheral blood in inflammatory bowel disease, IBD) [8]. As well as sample source, the granularity of expression studies can vary, from bulk sequencing of mixed cells, through sorted cell populations to single cells (with reduced sample size tending to associate with increased granularity). This is important because many signals have shown to be context dependent, so depth and breadth are required to detect cell state specific signals [9]. Other explanations include that non-coding variants may have regulatory functions beyond gene expression, and including spliceQTLs has been shown to increase the rate of colocalisation [10]. It has also been suggested that eQTLs and GWAS variants have other systematic differences, with eQTLs particularly enriched in promoters and typically situated much closer to genes, in addition to differences in functional annotations [4]. While this analysis holds for steady-state canonical eQTLs, other modalities differ. For example, enhancer RNAs (eRNAs) tend to have more distal eQTLs compared to canonical genes, a profile more similar to GWAS hits [11]. Indeed, including eRNAs along with canonical gene eQTLs led to a 63% increase in the proportion of GWAS signals showing colocalisation.

For a GWAS practitioner wanting to use molecular eQTL studies to aid interpretation of their new GWAS results, or for an investigator deciding whether to invest in a new eQTL study, it can be unclear which source of eQTLs are most likely to yield interpretable results. Here, we explore two massively parallel colocalisation analyses, one across diverse GWAS traits and eQTL cell types using results from Open Targets Genetics, and a second focused on immune-mediated diseases (IMD) and immune cells, in order to examine the dependence of the colocalisation gap on the properties of eQTL studies. Using this information we provide recommendations for the optimal eQTL study design to maximise colocalisations. In addition, while most signals colocalised with only one gene, we also observed colocalisation with two or more genes, similar to previous reports [1,2]. This occurrence hinders interpretation with regards to identifying the causal gene(s) and relevant cell type(s). We performed simulations and integrated promoter enhancer interaction data [12] with colocalisation results to investigate whether these multiple colocalisations were likely to represent false positives or co-regulation of neighbouring genes.

## Results

### 50% of GWAS signals in the Open Targets Genetics (OTG) resource colocalise with at least one gene

We began by considering the approximately 1.3 million colocalisation tests available in Open Targets Genetics (OTG). These data consist of pairwise comparisons of up to 126 eQTL datasets with 18,126 eGenes from 100 tissues or cell types and 4,687 GWAS datasets covering 24 categories of disease or traits, leading to 159,585 fine mapped GWAS peaks. This dataset represents the largest available public massively parallel set of colocalisation analyses to our knowledge.

**Fig 1:**
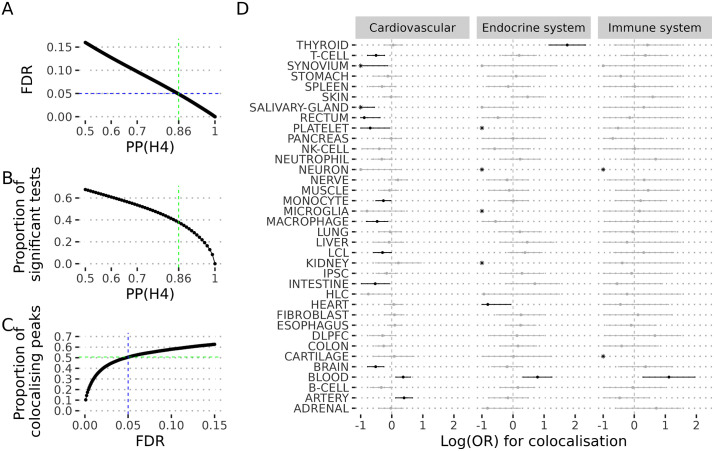
OTG colocalisation gap. **A.** Estimated false discovery rate (FDR) (y-axis) as a function of the posterior probability for colocalisation (PP(H4)) (x-axis). **B.** Proportion of colocalisation significant tests (y-axis) as a function of the posterior probability of colocalisation (PP(H4)). **C**. Proportion of peaks with evidence of colocalisation. In A,B and C, the vertical and horizontal lines indicate the thresholds selected from downstream analysis that correspond to 5% FDR. **D**. Log(OR) for colocalisation relative to adipose tissue and 95% adjusted credible intervals by the number of comparisons (x-axis) in different cell types or tissues (y-axis) for three exemplar disease traits. Black symbols indicate significant enrichment. The X axis is truncated at -1, and effect sizes less than -1 are shown by “*”.

Coloc examines genetic association patterns for two traits within a genomic region and evaluates whether they are consistent with a shared causal variant. It tests five hypotheses: **H0**, neither trait is associated; **H1/H2**, only one trait is associated; **H3**, both traits are associated but with different causal variants; and **H4**, both traits share the same causal variant. The number of significant coloc tests is a function of the threshold, α, on the posterior probability of colocalisation, PP(H4), used to call a significant test (Fig 1A). If PP(H4) is well calibrated, the expected false discovery rate (FDR) amongst all tests with PP(H4)> α is simply the mean of (1-PP(H4)) amongst that set of tests. We chose α as the smallest value for which the estimated FDR < 0.05 (Fig 1B), giving a value of α = 0.86. At this threshold, we found that overall 50.5% (95% confidence interval: 50.3-50.8%) of loci colocalised with at least one gene, with a median of 33% per trait (Fig 1C). Throughout the manuscript, “significant colocalisation” refers to results meeting this 5% FDR criterion.

### Colocalisation is more likely in disease-relevant cell types

To explore whether colocalisation rates differ depending on disease-relevance of the eQTL cell type, we selected all colocalisation tests for GWAS from three of the OTG categories: “immune system disease” (3,144 tests), “endocrine system disease” (8,331 tests) and “cardiovascular disease” (30,111 tests) for which we could identify likely causal tissues or cell types. All showed enrichment in biologically relevant eQTL datasets: immune system disease in blood, cardiovascular disease in artery and blood, and endocrine system disease in thyroid and blood (Fig 1D). This analysis supports another common approach to colocalisation analysis, focused on *a priori* likely causal cell types. To investigate this design, we considered a more specific set of colocalisation analyses, focused on immune-mediated disease (IMD) GWAS and their biologically relevant immune cell eQTLs. Immune cell eQTLs offer an opportunity to explore a rich diversity of eQTL study designs, and we assembled a catalogue of 12 eQTL studies (five not available in OTG) covering a total of 101 eQTL experiments from peripheral blood cells (S1 Table). This collection allowed us to consider the importance of the eQTL granularity - whether mixed cell populations with their attendant larger sample sizes could be more useful than smaller, sorted or single cell populations: our catalogue combined very well powered whole blood eQTL studies (eg eQTLGen [13] with sample size N > 30K), with sorted and single cell eQTL studies on a wide range of cell types (>20 cell types for ImmunexUT [14] or OneK1K [9], albeit with N under 1000). While most studies were of European ancestry, ImmunexUT participants were of Japanese ancestry while other studies combined individuals of European and Asian [15] or African [16] ancestry. We also addressed whether samples from individuals with IMD may be more informative than those from healthy controls [7] by including eQTL studies combining IMD donors and controls or stimulated cells (S1 Table and S1 Fig).

**Fig 2:**
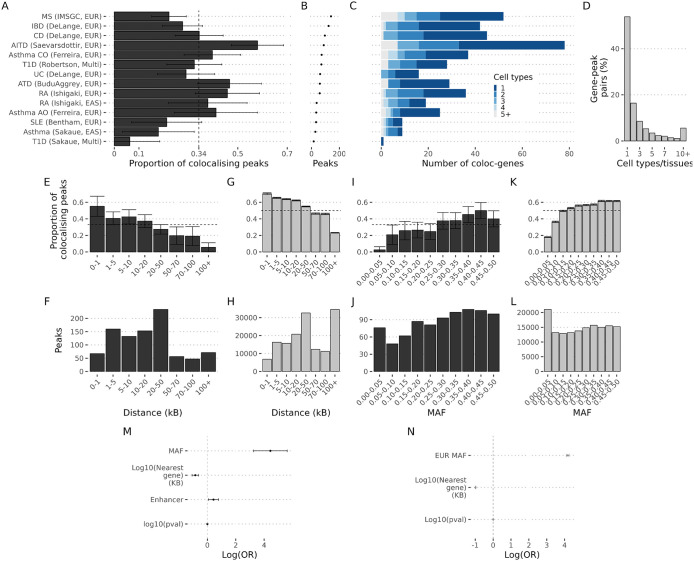
Colocalisation gap by IMD and characteristics of GWAS lead variants stratified by colocalisation status. **A-C,E-F,G,I-J and M correspond to the analysis of IMDs. D,G-H,K-L and N correspond to OTG**. **A**. Proportion of GWAS loci with at least one significant coloc test (bar plot) for each IMD. The dashed vertical line represents the average proportion, 0.34 [95% CI 0.30 - 0.36]. **B**. The dot plot shows the total number of GWAS loci by IMD. **C.** Cell specificity of colocalising genes. eQTL studies were aggregated by nominal cell types (CD4 T, CD8T, double negative T, gamma delta T, Monocyte, Neutrophil, B, DC, NK, HSPC, MAIT, Platelet). For each gene colocalising with a peak, the number of cell types that the colocalisation was detected was recorded. The plot shows the number of colocalising gene-pairs stratified by the number of cell types detected (x-axis) by IMD (y-axis). **D**. For each gene colocalising with a GWAS peak in OTG we counted the number of cell types or tissues that a significant colocalisation was observed. The plot shows the proportion of gene-peak pairs detected in the indicated number of cell types or tissues (x-axis). The plot was truncated at 10 cell types or tissues. **E.** The GWAS lead variants from the IMD analysis were aggregated by the distance to their nearest TSS gene in the bins shown in the x-axis. For each bin, the proportion of peaks with at least one significant coloc test was calculated (y-axis). The error bars correspond to the 95% confidence intervals. **I.** The plots show the total number of GWAS peaks for the analysis of the IMDs (y-axis) for each of the intervals (x-axis). **G-H.** Same as E-I except that the analysis corresponds to the GWAS lead variants from OTG. **I-J,K-L**.Similar to E-I except that GWAS lead variants were aggregated by their minor allele frequency in the bins shown in the x-axis. I-J corresponds the analysis for IMDs, and K-L to OTG. **M**. The plot shows the log odds ratio for colocalisation in the IMD study for the minor allele frequency (MAF) of the GWAS lead variant, the log based 10 for the distance in kilo base pairs between the GWAS lead variant and the nearest gene (Log10(distance to nearest gene (KB)), whether the GWAS lead variant overlaps and active enhancer in immune cells (Enhancer) of the log based 10 for the p-value of the GWAS variant. **N**. Log odds ratio for colocalisation in the OTG study for European minor allele frequency, distance to nearest gene and log in base 10 for the trait association and log based 10 for the GWAS p-value.

We colocalised eQTLs from each of these datasets with 14 exemplar IMD GWAS covering 10 distinct IMDs (S2 Table). We performed 153,656 colocalisation tests, related to 832 GWAS peaks (defined as containing a credible set in OTG fine mapping analysis of the same dataset, see Methods). We also included six non-IMD diseases (S2 Table) for comparison for which we conducted 65,272 additional coloc tests. We again applied a 5% FDR to define significant colocalisations, which corresponded to α = 0.88 (S2A – S2B Fig). We found that 34% of GWAS peaks colocalised with at least one eQTL (Fig 2A and S3 and S4 Tables), lower than the estimate from the broader OTG study (50%, Fig 1C), possibly due to the narrower cell type coverage in this focused design which does not capture non-immune cell elements of disease aetiology, such as beta cell function in type 1 diabetes. Otherwise, colocalisation rates were not significantly related to the power of the GWAS study (Fig 2A and 2B) and were higher for IMDs (when the cell types used in the eQTL studies play a role in the disease process) than when compared with a set of non-IMD GWAS (S2C Fig), with an odds ratio for IMD colocalisation of 1.6 (1.4-2.3), relative to non-IMDs. We found that protein coding genes alone were able to explain ~90% of the GWAS peaks with a colocalisation, while the remaining 10% could only be explained by non-coding RNAs.

We then compared the properties of the lead GWAS variants (as proxies for the causal variants) with or without evidence of colocalisation both for IMDs and OTG. Mostafavi et al [4] recently explored the absence of colocalisation identified for many GWAS signals, describing significant differences in the patterns of GWAS and eQTL association signals. In agreement with this, we found that the proportion of GWAS peaks with evidence of colocalisation consistently decreased as the distance between the lead GWAS variants and their proximal gene transcription start site (TSS) increased both in the IMD and OTG analysis (Fig 2E - 2H, 2M, and 2N). Moreover, we also saw that the proportion of GWAS peaks with evidence of colocalisation increased as the minor allele frequency of the GWAS lead variants increased (Fig 2I - 2L, 2M, and 2N), suggesting a lack of power to detect potential eQTLs for lower-frequency variants in current eQTL studies. In addition, colocalising IMD GWAS lead variants tended to be within predicted active enhancers for PBMCs (Fig 2M). The nearest gene to a GWAS peak is usually considered the most likely causal gene, supported by our results in IMDs which linked 65% of significant colocalisations to the closest gene when considering protein coding genes only or 50% for all genes (S2D Fig).

### Colocalisation enrichment in more granular designs though studies still underpowered

**Fig 3:**
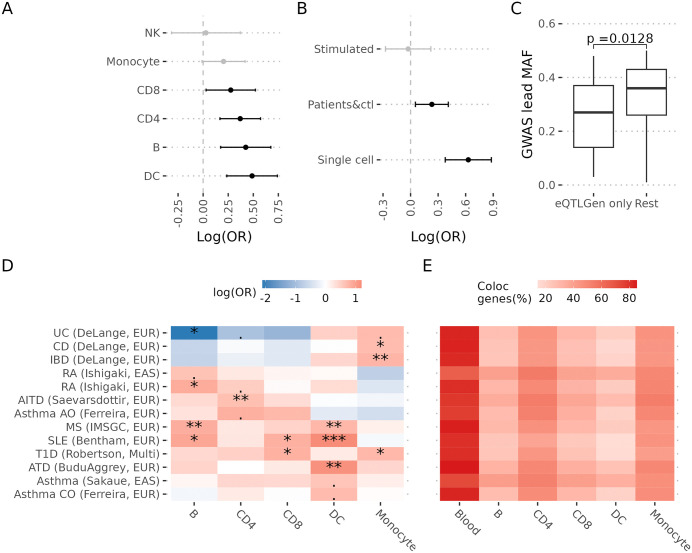
Trade-off of different eQTL designs. This analysis was performed for IMDs. **A**. Colocalisation odds ratio (x-axis) for the indicated cell types (y-axis) relative to whole blood. **B.** Colocalisation odds ratio (x-axis) for single cell eQTL studies relative to sorted cell, stimulated relative to steady state and mixture of patients and controls (Patients&ctl) relative to controls. **C**. Allele frequency distribution for the GWAS SNP (y-axis) stratified by those which only showed significant coloc tests in eQTLGen vs those detected in any other eQTL experiment (rest). **D.** Heatmap showing the enrichment for colocalisation for the indicated cell types (x-axis) relative to whole blood stratified by IMD (y-axis). The symbols indicate significance level (0.05 ⪯ p < 0.1, * 0.01 ⪯ p < 0.05, ** 0.001 ⪯ p < 0.01, *** p < 0.001). **E**. Heatmap showing the proportion of coloc significant genes identified by each of the indicated cell types (x-axis) for the IMDs in the y-axis.

We exploited the diversity of our IMD-focused panel of colocalisation tests to assess how eQTL study design impacted colocalisation discovery. We first tested for enrichment in the proportion of colocalisation significant tests within nominal immune cell types, that is, the chance of colocalisation conditional on detecting an eQTL. Relative to whole blood, CD8 and CD4 T cells, B cells and dendritic cells showed significant enrichment, as did single cell eQTL datasets relative to sorted cells, and eQTL experiments with a mixture of patients and control samples, relative to control samples only, (Fig 3A - 3B). In contrast, the sample of stimulated cells we considered did not show enrichment (Fig 3B). The lower rate of colocalisation per whole blood eQTL compared to most sorted or single cell eQTLs, is compensated by the larger sample size afforded by meta-analysis (over 30,000 samples) which leads to increased eQTL discovery: more colocalising genes are found in eQTLGen than any other dataset (S3 Fig), and eQTLGen is often the only source of colocalisations for GWAS peaks with rarer variants (Fig 3C), suggesting the lack of power associated with smaller sample sizes for more sophisticated designs is an important issue [17]. It is important to note, however, that using only eQTLGen or even all blood samples would not be sufficient: 48% of GWAS peaks that showed colocalisation only did so using sorted or single cell eQTL studies, compared to 15% that showed colocalisation only in whole blood.

We also saw considerable evidence of cell-type specificity. In OTG, with a broad range of tissues, we found that 53% of the peaks with a significant colocalisation test with a particular gene were detected in only one tissue/cell type (Fig 2D). In our IMD study, we found that 55% of colocalising genes were detected in one nominal cell type only (CD4 T, CD8 T, double negative T, gamma delta T, MAIT, Monocyte, Neutrophil, B, DC, NK, HSPC, Platelet), with a rapid decrease in the number of genes colocalising in multiple cell types, a pattern that was shared across IMDs (Fig 2C). Additionally, we found specific patterns of colocalisation enrichment across IMDs, recapitulating known biology. For example, Crohn’s disease and inflammatory bowel disease colocalisation signals were enriched in monocytes, while systemic lupus erythematosus colocalisation signals were enriched in B cells and dendritic cells (Fig 3D). However, when we looked at the proportion of colocalisations by cell type/tissue in each IMD, whole blood was the predominant contributor across the board, reflecting again the increased power of eQTLGen (Fig 3E).

### Genes colocalising with the same GWAS peak show evidence for co-regulation

**Fig 4:**
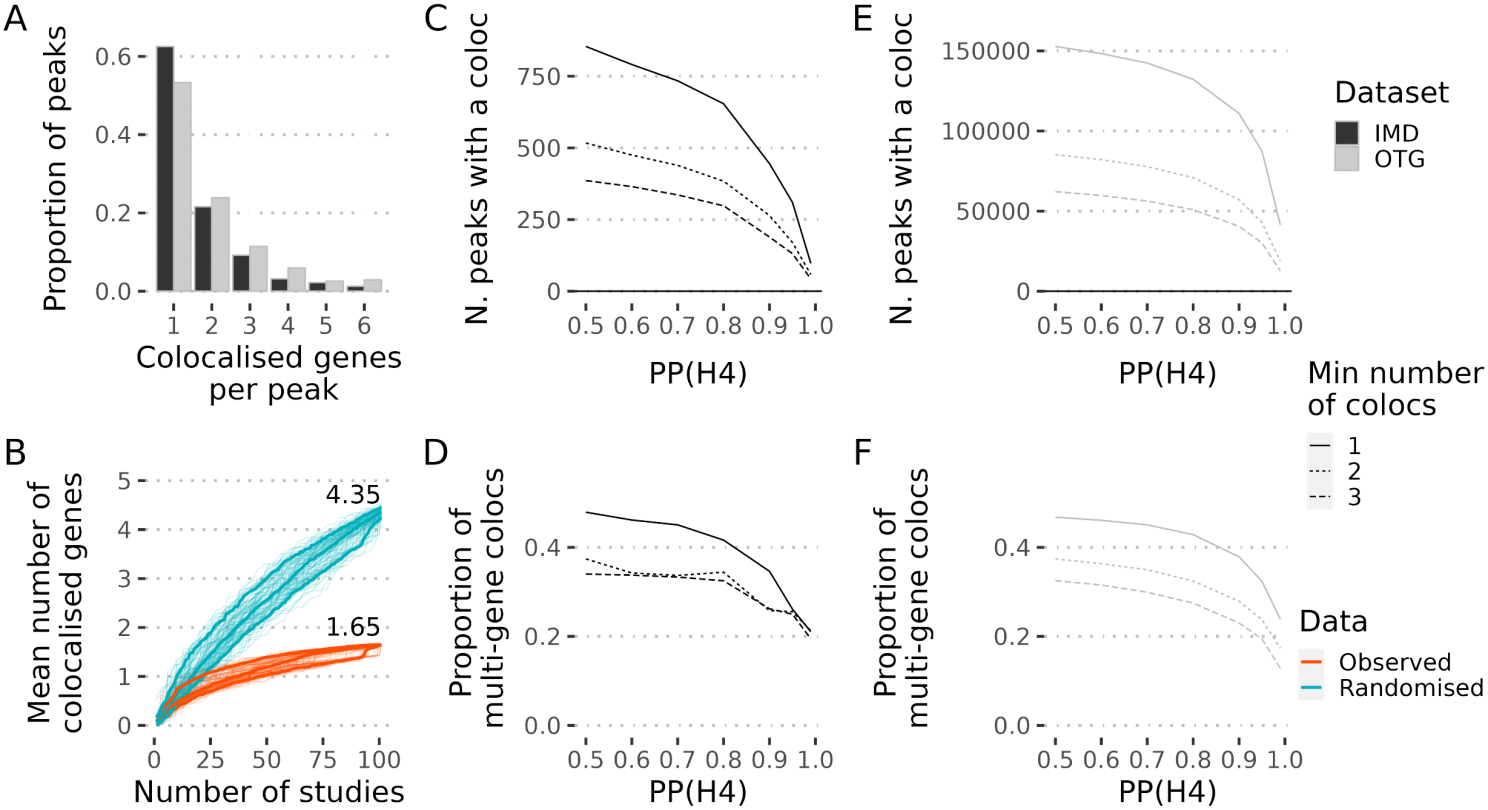
Assessing false positive colocalisation calls within GWAS peaks with multiple colocalising genes. **A.** Distribution of the proportion of colocalised genes per peak in the IMD (black bars) or OTG (grey bars) analysis. **B.** Cumulative mean of the number of colocalised genes after adding each study. The order of the 101 eQTL studies was randomised 100 times. In each iteration the cumulative mean of the colocalised genes across all peaks was calculated after the addition of each study, either with the observed data (orange) or on previously randomised PP(H4) within each peak (blue). Each iteration is represented by a fine line. The thicker lines correspond to the 10, 50 and 90% quantiles. The numbers in the plot correspond to the median of the iteration means after the addition of all the studies. **C,E.** At each of the indicated thresholds for the posterior probability of H4 (x-axis), the number of peaks with at least 1 (solid line), 2 (dotted line) or 3 (dashed line) colocalising genes is represented is shown for the IMD analysis (C) or OTG **(E)**, respectively. **D,F.** Similar to C,E except that the fraction of multi-gene colocalisations across peaks is shown for the IMD analysis (D) or OTG **(F)**, respectively.

We found that ~47% of GWAS peaks with significant colocalisation in OTG and 37% in the IMD analysis displayed this colocalisation with two or more genes, though the number of peaks with more than two colocalising genes rapidly decreases (Fig 4A). Among the peaks with multiple colocalising genes in the IMD analysis, approximately half (60/118) colocalised with protein-coding genes only, 47% (55/118) colocalised with both protein-coding and non-coding RNA genes, and the remaining ~3% (3/118) colocalised exclusively with non-coding RNA genes.

One explanation for the number of peaks colocalising with multiple genes is that coloc is failing to control the false discovery rate, so that with increasing numbers of eQTL datasets we find more colocalisations, and that the multiple colocalisations represent false discoveries. We compared the observed mean number of colocalising genes per GWAS peak to that expected under a model of random colocalisation using the IMD analysis. False discoveries should be distributed randomly across tested genes, but we found that observed colocalisation signals were significantly concentrated within the same genes in the observed data (Fig 4B). We also examined how the number of GWAS peaks with multiple colocalising genes changed as a function of increasingly stringent thresholds for calling colocalisation (Fig 4C and 4E). Even when increasing stringency to the point of reducing the number of GWAS peaks with colocalisations from 854 to 97 in the IMD analysis or from 152K to 41K in OTG, the fraction with multiple colocalising genes remained above 20% for IMD or 15% for OTG (Fig 4D and 4F). While we cannot rule out false colocalisations, and indeed expect 5% of our calls will be false given we targeted 5% FDR, these findings suggest that false positives alone cannot explain the multi-gene colocalisations.

Alternatively, they could reflect distinct GWAS signals that were merged to form index GWAS peaks (see Methods). However, the proportion of peaks with two or more colocalising genes was similar (37% vs 38%) when we excluded from the analysis peaks resulting from merging GWAS signals defined by Open Targets. Another possibility is that errors in the alignment of RNA-seq reads for genes with high sequence similarity, which tend to be in close proximity, may appear to have correlated expression and lead to apparent multi-gene eQTL signals in a locus. We considered the pre-computed “cross-mappability” indices between every pair of annotated genes in the human genome by Saha et. al. [18]. We did not observe a significant difference (p = 0.7) between the proportion of gene pairs with no evidence of mappability issues in pairs of genes colocalising with the same peak (0.84; 80/95 pairs) and pairs of colocalising and non-colocalising genes tested at the same GWAS signals (0.81; 77/95). Excluding genes with evidence of mappability issues led to only a small increase in the proportion of peaks with only one colocalising gene in both datasets (0.63; 197/315 to 0.65; 202/311 in IMDs, 0.53; 42766/80246 to 0.59; 47549/80223 in OTG, S4 Fig). We concluded that mappability errors are not a major driver for the patterns of muti-gene colocalisations.

**Fig 5:**
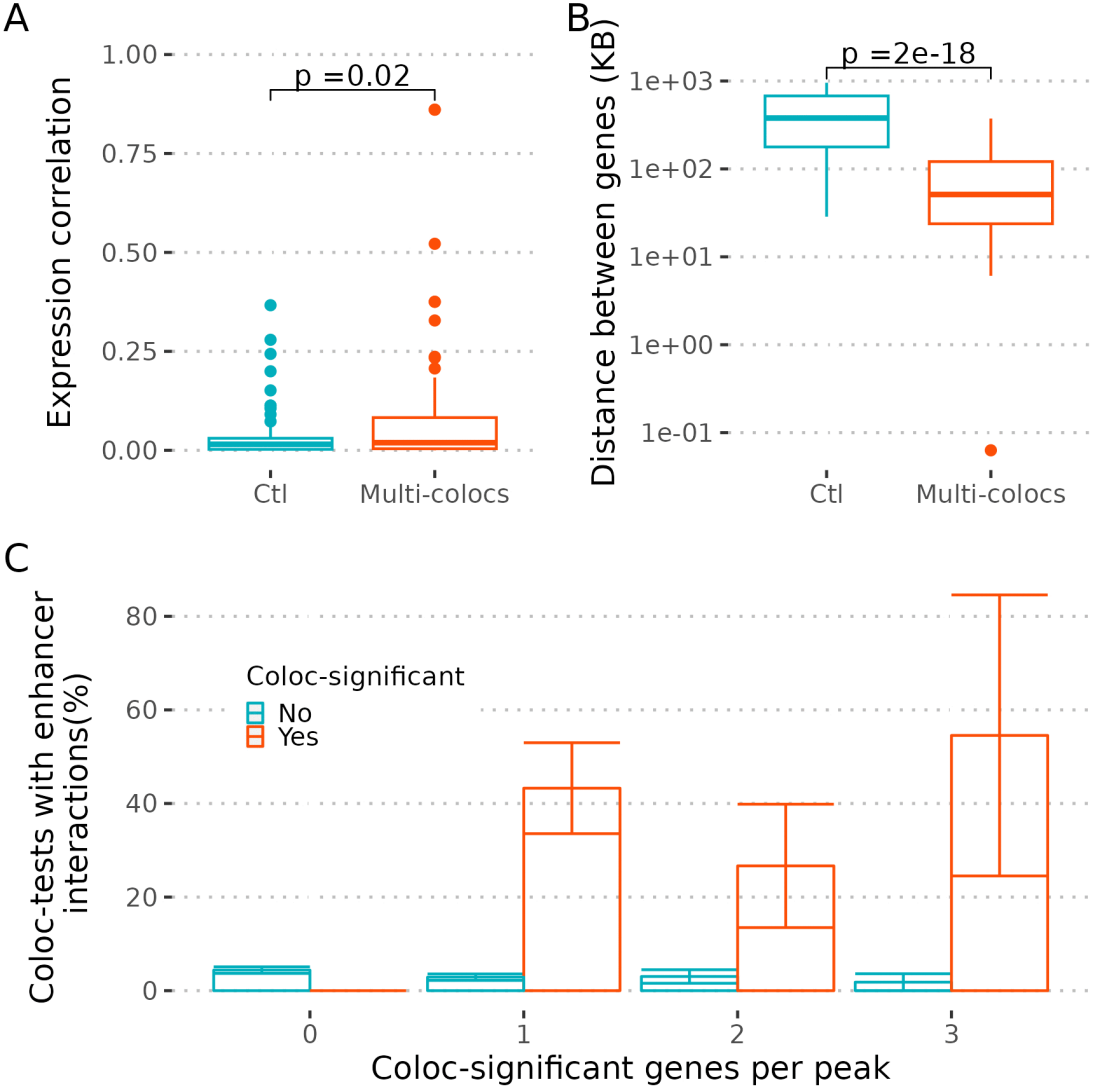
Evidence for co-regulation of multi-coloc genes. **A**. Here, for each eGene we selected the eQTL study with the highest PP(H4) for a given peak. Then, for each pair of significant colocalising eGenes a control pair was designed by randomly selecting one of the two colocalising eGenes and making a new pair with the selected eGene and a non-significant eGene tested with the same peak and expressed in the same cell type of the non-selected eGene. The non-significant eGenes were only selected once. This procedure gives the same number of tests and control pairs while matching cell types. The plot shows the distribution of the squared expression correlation (y-axis) for multi-gene colocalisations and controls (x = axis). The p-value for the mean difference is indicated. **B**. Using the same groups as in A, the distance between the TSS of the genes in a pair was calculated and the distribution is shown. The p-value for the mean difference is indicated. **C.** We selected GWAS peaks with the lead variant overlapping an enhancer in immune cells (21%). eQTL studies were aggregated by cell types matching the cell types with enhancer annotations (monocytes, dendritic cells, CD4 T, CD8 T, NK and B cells). Then, for genes with multiple coloc tests within the aggregated cell types, we selected the coloc test with the maximum PP(H4). Next, we stratified peaks by the number of colocalisation significant genes (0-3). For the peaks with 1 or more significant colocalising eGenes, we stratified the coloc tests by significance (blue/orange). Last, for each group of coloc tests we calculated the proportion of predicted enhancer interactions with the gene promoters matching the cell type of the eGene and the enhancer, which is shown in the y-axis.

An alternative explanation is that peaks with multiple colocalising genes reflect genuine shared regulation, a hypothesis supported by evidence that proximal genes may be co-expressed and share regulatory elements [19–21]. Excluding blood (a mixed tissue), we identified 549 non-overlapping GWAS peaks where at least two genes were tested for colocalisation (see Methods). At each peak we considered all pairs of colocalisation tests formed from distinct genes. We found that both tests were more likely to be significant colocalisations if they were from the same cell type compared to different cell types (odds ratio = 1.2, p = 0.004). Despite this, when different genes did colocalise with the same GWAS peak, the average squared correlation between the lead eQTL SNPs did not differ between eQTLs colocalising with the same gene in the same or different cell types (mean r^2^ same = 0.84, mean r^2^ different = 0.84, p = 0.99; calculated in European studies). Pairs of genes colocalising with the same peak tended to show higher correlation in expression, and to be closer to each other, relative to pairs of colocalising and non-colocalising eGenes tested at the same GWAS signal (Fig 5A-5B).

**Fig 6:**
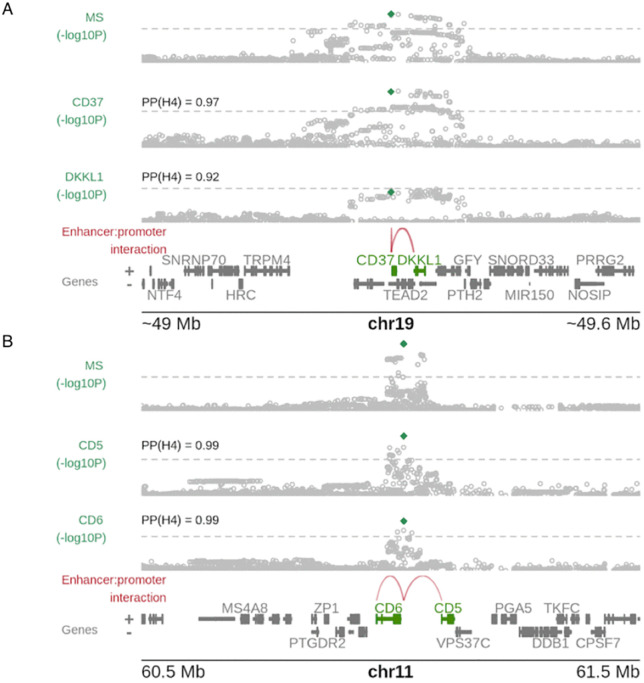
Examples of multi-coloc genes. **A**. Genomic region for MS signal in chromosome 19. The top panel shows the Manhattan plot for MS. The second and third panels show eQTL plots for *CD37* and *DKKL1* from B cells (OneK1K and Schmiedel), respectively. The posterior probability for the colocalisation hypothesis is indicated (PP(H4)). The dashed grey line corresponds to the genome wide significant level (p = 5x10^-8^). The green symbol corresponds to the lead GWAS SNP. The interactions between the enhancer overlapping the lead GWAS and the gene promoters is indicated by the red arcs. The genes in the region are depicted in the figure, with *CD37* and *DKKL1* in green. **B.** Same as A but for the MS peak in chromosome 11 that colocalised with *CD5* and *CD6* in CD8 T cells. The eQTL plots are from naive CD8 T cells from the ImmunexUT study.

Lastly, we tested whether colocalising genes could be independently linked to their colocalising GWAS peaks, where those GWAS peaks overlapped an enhancer, based on the publicly available maps for enhancer-gene interactions in immune cells using the scE2G model [12] (Methods). We found that 40% of GWAS lead SNPs with at least one colocalising eGene were located in enhancers linked to their significant eGenes, compared to 3% for GWAS lead SNP/eGene pairs which did not colocalise (p = 10^-10^). The proportions were similar whether we considered all genes together, or stratified by the number of colocalising genes per peak (p = 0.9, Fig 5C), arguing against multiple-colocalising genes being all false positives. Forty of the GWAS peaks that overlapped active enhancers colocalised with expression of two or more nearby genes. For example, a multiple sclerosis (MS)–associated peak on chromosome 19 lies proximal to *CD37* and *DKKL1*. The overlapping enhancer is predicted to contact the promoters of both genes in B cells, and colocalisation analysis indicates that the MS signal colocalises with cis-eQTLs for *CD37* and *DKKL1* in B cells (Fig 6A). The two genes show modestly correlated expression in B cells (Pearson *r* = 0.20, *p* = 0.001). Functionally, *CD37* is implicated in B-cell survival and proliferation, whereas *DKKL1,* expressed at low levels in B cells, is principally known to regulate Wnt signalling, and shows high expression in testis. Although both genes are linked to the same regulatory region, current evidence does not establish whether both influence MS risk; it remains plausible that only one is causal while the other is a correlated target of the enhancer.

A second example is an MS-associated peak on chromosome 11 near the paralogous genes *CD5* and *CD6*. The enhancer overlapping this peak is predicted to interact with both promoters in CD8 ⁺ T cells, and eQTL colocalisation provides strong support that the GWAS signal and expression of *CD5* and *CD6* share a causal variant in CD8 ⁺ T cells (Fig 6B). Although co-regulated genes do not necessarily display correlated expression (they may share only some subset of enhancers), *CD5* and *CD6* are highly co-expressed in CD8 ⁺ T cells (Pearson *r* = 0.70, *p* < 10 ⁻ ⁶), which also suggests coordinated regulation by shared regulatory elements; both genes function in T-cell receptor signalling. Although it is currently unknown whether these genes influence MS risk, given the shared functionality, it may be possible that both are causally related.

## Discussion

Although colocalisation was originally devised to be deployed in a semi-hypothesis driven setting, integrating lipid trait GWAS with liver eQTLs [22], it is now often deployed in a massively parallel approach, taking all available molecular QTL studies compared to one or many GWAS. In this study, we characterised the results of two massively parallel colocalisation analyses, with a goal to make recommendations as to the optimal selection and design of eQTL studies to maximise candidate effector gene discovery by colocalisation. Colocalisation with coloc can be run in two modes: either under a single causal variant assumption or allowing for multiple causal variants by fine mapping with SuSiE [23] first. The former approach is computationally faster, and avoids the need to carefully match reference LD matrices to each GWAS and eQTL dataset, while the latter is likely to discover more colocalisations. Both studies here used coloc with a single causal variant assumption, therefore our focus in this analysis was the relative detection of colocalisation between different eQTL study designs rather than the absolute number of colocalisations. The new Open Targets Platform (https://platform.opentargets.org/) includes a fine mapping step where possible, which would allow a comparison with single and multiple causal variant approaches in the future.

Sample size was a key determinant of the number of colocalisations in agreement with recent findings [17]. Indeed, the study with the largest sample size and lowest granularity - eQTLGen - enabled the largest total number of colocalisations to be detected. Despite this, eQTLs in higher-granularity studies were more likely to colocalise, and over 50% of colocalisations were specific to one tissue or cell type. This agrees with findings of the TenK10K study [24] which found that using cell-type eQTL increased the number of colocalisations by 5.2x compared to a whole dataset pseudobulk eQTL analysis. We also saw an increased chance of colocalisation in disease-relevant tissues and samples that included patients. This agrees with a recent study which was able to colocalise 56% of IBD GWAS loci using single-cell eQTLs mapped in a cohort of 421 individuals, including both blood and intestinal biopsy samples from both healthy controls and individuals with IBD, substantially more than the 43% reported in OTG [8]. Here, again, eQTLs identified at the cell-type level were over two-fold likely to colocalise compared to those identified at low resolution, consistent with cell type specific eQTLs being more distal to genes and located within enhancer regions [8], and therefore more similar to GWAS signals.

Our results suggest the optimal approach for colocalisation is to maximise both granularity and sample size. However, granularity and sample size were inversely correlated in the eQTL studies we considered, reflecting the relative cost of sequencing bulk tissue, sorted cells and single cells. Therefore, we recommend practitioners should select eQTL studies covering a diverse set of disease-relevant cell types and contexts at high granularity, preferably from single cell studies. These high granularity studies should be complemented by higher sample size studies, even of lower granularity.

The difference in allele frequency profile between colocalisations detected with eQTLGen and other high granularity studies is evidence that more colocalisations should be detectable with higher granularity studies if we could increase sample size. One option is through meta-analysis of eQTL studies, as in eQTLGen used here and also available for adipose tissue [25], although care needs to be taken about carefully matching cell type definitions to avoid obscuring the cell-specific nature of colocalisations. However, several large-scale single cell eQTL studies are in progress, which will provide both high granularity and high sample size resources in peripheral blood mononuclear cells in the near future. The TenK10K project aims to profile 10,000 individuals and has already collated data from 1,925 [24], while a recent study in 1,108 Finnish individuals showed that eQTLs can be paired with chromatin accessibility to increase colocalisation [26]. It is hard to see how either large single cell studies or very large eQTL meta-analyses of sorted cells will be achieved in patient populations, though the more modest single cell studies in a few hundred individuals are likely to be invaluable to access cells from a disease context. As scientists become more familiar with genome language models such as Borzoi [27] and AlphaGenome [28], and as such models become more accurate, it may be that these can be used to overcome sample size limitations and provide context-dependent inference of eQTLs with manageable numbers of samples from the relevant context. Finally, other important directions to close the colocalisation gap, not examined here, include extending the molecular QTL catalogue to include protein, eRNA, splicing and chromatin accessibility eQTLs.

While increasing the number of cell types studied increases the chance of a GWAS signal colocalising with at least one eQTL, we also saw a concomitant increase in the average number of colocalised genes per GWAS signal. With multiple testing, there is always the risk of increasing the number of false positives with increasing tests, which is why we used an FDR-based threshold to declare colocalisation. We note that the thresholds estimated in each of these large studies, 0.86 and 0.88, are higher than those typically used in colocalisation analysis. The optimal threshold will depend on the downstream consequences of “calling” a colocalisation. If there is a need to limit false discoveries, then we would advocate the use of an FDR-based approach to determining a threshold. But if colocalisation is a screening step towards designing a large-scale molecular experiment, then a more liberal threshold may be appropriate to ensure true positives are not missed. As ever, there is no one size fits all, and we encourage practitioners to think carefully about the appropriate threshold for their circumstances.

Our initial concern seeing multiple genes colocalising with the same GWAS signal was that they may reflect inadequate control of false positives. Indeed, methods have been developed to identify the single causal gene in the presence of multiple colocalisation signals, under the assumption that only one gene can be causal [29]. However, sensitivity analysis suggested that even very stringent thresholds did not substantially lessen the issue in either of the studies we considered. Instead, we observed evidence suggesting co-regulation of these “multi-coloc” genes could be at least part of the explanation. Co-regulation of multi-coloc genes clearly complicates interpretation, because only one of the genes may be on the causal pathway, with the remainder acting effectively as biomarkers for the disease-risk variant, and possibly for the causal mechanism [19]. An example is provided by the MS-associated GWAS peak that colocalises with *CD37* and *DKKL1* in B cells. Although it remains unclear whether either gene is causally linked to MS, their weak expression correlation and apparent involvement in distinct signalling pathways suggest divergent biological functions, making it plausible that only one gene is directly causal. On the other hand, multiple colocalising genes may act in concert, with both contributing to disease pathogenesis. It is possible this pattern underlies the colocalisation of the paralogous genes *CD5* and *CD6*, both key regulators of T-cell receptor signalling, with another MS-associated signal.

In the case of co-regulation, observational studies are insufficient to disentangle causality. Experimental perturbations of the expression of each candidate causal gene performed individually, for example using CRISPR-cas9 based approaches, combined with molecular readouts will be required to determine which gene(s) mediate the effect. One possible strategy to guide these experiments is to construct transcriptional networks from healthy and patient derived samples to infer whether the signalling pathways regulated by the multi-coloc genes converge in a downstream process and what differences can be inferred between patients and controls. Such network analysis could guide the selection of molecular targets and readouts for experimental perturbation at the cellular level. Finally, our analysis also reveals that a considerable number of non-coding RNAs colocalised with disease associated variants, highlighting that we remain in the early stages of functionally characterising these regulatory transcripts and their potential contribution to immune-mediated disease. Non-coding RNAs may be co-regulated with nearby protein-coding genes, and it may be that any disease association is with the protein-coding gene, even if colocalisation was not detected. However, there is also emerging evidence that lncRNAs can also affect transcription and translation of distant genes, can serve as scaffolding facilitating protein-protein interactions, act as miRNA sponges and therefore regulate miRNA activity [30], such that lncRNAs could affect disease risk in their own right.

Another consideration in this new paradigm, where more than one gene frequently colocalises with a single GWAS peak (this study, GTEx [1] and OTG [31]), is that conclusions for prioritising genes for functional follow-up studies may vary depending on the sources and scope of molecular QTL data used. For instance, in our study, we observed that an MS signal in chromosome 19 colocalised both with *CD37* and *DKKL1* in B cells. A previous study nominated *DKKL1* as the putative causal gene based on colocalisation with a pQTL [32]; however, that platform did not include CD37. In contrast, yet another study prioritised *TEAD2* for the same MS locus by integrating eQTL data with promoter capture Hi-C (PCHiC), which identified an open chromatin region in CD4 ⁺ T and B cells overlapping the lead GWAS variant and interacting with the *TEAD2* promoter [33]. In this case, the GWAS peak SNP was too close to the CD37 transcription start site (~7 kb) to assess promoter interaction. Note that we also see colocalisation with *TEAD2*, but only in whole blood. This example underscores the importance of integrating multiple data types while carefully considering biases and data gaps that may influence causal gene prioritisation. In particular, the “denominator” set of genes that are considered in an experiment, often defined according to interest in a specific disease, may create blind spots which should be acknowledged when positive findings are reported. We suggest that triangulation of evidence is going to become increasingly important to prioritise causal genes as the breadth of molecular data continues to grow.

## Materials and methods

### OTG colocalisation datasets

We downloaded data from Open Targets Genetics, files now archived at https://ftp.ebi.ac.uk/pub/databases/opentargets/genetics/latest/v2d/.

### GWAS datasets

We collected 22 publicly available, well-powered GWAS datasets (minimum N cases = 5,201), including eight immune-mediated diseases (IMD): autoimmune thyroid disease [AITD] [34], atopic dermatitis [ATD] [35], asthma [36], inflammatory bowel disease [IBD] [37], multiple sclerosis [MS] [38], rheumatoid arthritis [RA] [39], systemic lupus erythematosus [SLE] [40] and type 1 diabetes [T1D] [36,41]. This included two asthma subtypes (child-onset [Asthma CO] and adult-onset [Asthma AO]) [[42]] and two IBD subtypes (Crohn’s disease [CD] and Ulcerative colitis [UC]) [37]. To compare discoveries in immune and non-immune-mediated traits, we included three psychiatric/neurological disorders (Alzheimer’s [43], major depression disorder [MDD] [44] and schizophrenia [SCZ] [45]), and three non-immune-mediated conditions (myocardial infarction [MyoInf] [36,46], osteoarthritis [OArth] [[47,48]] and type 2 diabetes [T2D] [33]). The ethnicity of the GWAS participants is described in S2 Table.

All datasets were processed using an in-house pipeline to ensure they all have GRCh38/hg38 coordinates and conform to a standard format with minimum requirements of allele, effect size, standard error, and p-value information for all SNPs (see github.com/GRealesM/GWAS_tools).

### eQTL datasets

We collected 101 publicly available blood-cell RNA-seq eQTL datasets from 12 studies [1,9,13–15,49–55] and 13 primary cell types: B cells, CD4 + T cells, CD8 + T cells, double-negative T cells (dnT), gamma-delta T cells (gdT), mucosal-associated invariant T (MAIT) cells, NK cells, dendritic cells (DC), hematopoietic stem and progenitor cells (HSPCs), monocytes, neutrophils, platelets, and whole blood. These datasets include bulk- and single-cell RNA-seq experiments from healthy volunteers and IMD patients of European, African, and Japanese ancestry, with sample sizes ranging from 82 to over 30,000. We also incorporated datasets of naive cells from healthy volunteers stimulated with viral and bacterial stimuli (S1 Table).

Most datasets were obtained from the eQTL Catalogue [55] and required minimal pre-processing. For the eQTLGen whole blood dataset [13], we used the UCSC liftOver tool to get the GRCh38/hg38 coordinates. For the ImmunexUT datasets [14] we used data from the Japanese (JPT) sample of 1000Genomes Project Phase III to estimate the allele frequencies. To allow cross-study comparisons we normalised effect sizes (β) and corresponding standard errors (SE) by the estimated standard deviation of the trait (${\hat{\sigma }}_{Y}$). Allele frequencies were used to estimate $\sigma_{Y}$ as ${{\hat{\sigma }}_{Y}}^{\text{2}}=\frac{\sum v_{i}w_{i}}{\sum {w_{i}}^{\text{2}}}$ where $w_{i}\;=\;\frac{\text{1}}{SE_{i}}$ $v_{i}=\;\text{2}nf_{i}(\text{1}-f_{i})$ and *n* is sample size, *f*_*i*_ is MAF of SNP *i* and SE_*i*_ is the standard error of the effect estimate, $\beta_{i}$ at SNP *i*.From these, we calculate corrected estimates and standard errors, ${\overset{―}{\beta }}_{i\;}=\frac{\beta_{i}}{\sqrt{\overset{\text{2}}{\underset{Y}{\hat{\sigma }}}}},\;{\overset{―}{SE}}_{i}=\frac{SE_{i}}{\sqrt{\overset{\text{2}}{\underset{Y}{\hat{\sigma }}}}}$.

To identify eGenes for testing, we took the lowest p-value in each gene, applied Bonferroni correction (i.e., lowest p-value × number of SNPs in that gene), and applied the FDR procedure to the resulting Bonferroni-adjusted p-values, selecting the genes with FDR < 1%.

### Colocalisation in IMDs and QC

We applied a colocalisation and QC procedure on each eGene across all eQTL-GWAS combinations (22 GWAS and 101 eQTL datasets). For each combination, we performed colocalisation when a GWAS dataset contained at least one significant (p < 5 x 10^-8^) SNP within the eQTL region (typically 2 Mb centered on the eGene TSS). We used the statistical package *coloc* [56] to assess the evidence of colocalisation. Coloc requires that input SNPs are present in both datasets, producing unreliable results if the strongest associated SNPs for one dataset are missing in the other. To control for this, we used the finemap.abf() function to compute single causal variant finemap posterior probabilities $p_{\text{1}i},\;p_{\text{2}i}$ for traits 1 and 2, and SNPs *i*. We calculated cross-trait summed posterior probabilities across the set of shared SNPs *S* as $q_{\text{1}}=\sum_{i\;\in \;S}p_{\text{1}i}$, $q_{\text{2}}=\sum_{i\;\in \;S}p_{\text{2}i}$, proceeding only if $min(q_{\text{1}},\;q_{\text{2}})\;>\;.\text{9}$, to ensure the inclusion of the most likely causal SNPs for both traits in the shared set *S*. This resulted in 221,993 coloc tests performed. We ran *coloc* under a single causal variant, with a conservative prior for the hypothesis of colocalisation to reduce the chance of false positive calls (p12 = 5e-6) [56]. To minimise spurious results, we discarded tests associated with deprecated genes in Ensembl (version 112).

### Significance threshold for colocalisation

We called significant tests those with FDR < 0.05, both in OTG and our in-house analysis. For each test, coloc outputs the posterior probability of colocalisation, PP(H4). We estimated the FDR as the mean of (1-PP(H4)) among the tests with PP(H4)> α and selected α as the largest value such that estimated FDR < 0.05.

### Definition of GWAS peaks

While colocalisation is performed on regions defined by the eQTL (typically 2 Mb around a gene TSS), it is helpful to annotate colocalisations with the GWAS peak for downstream analysis. We downloaded fine-mapping 95% credible set data from the OpenTargets platform for each GWAS dataset on 30/5/2025. We used the lead SNPs in each credible set (1478) as a representative of independent GWAS signals (henceforth named peaks) to link our coloc tests to for downstream analyses. For each test eQTL region, we checked whether it contained any GWAS peak for its corresponding GWAS dataset, assigning the peak to the test if only one was found. This was the case for 1329 peaks. For 29,472 eQTL regions (including 277 colocalisation-positive) which contained more than one peak, we visually inspected the multiple peak location in the context of GWAS and eQTL Manhattan plots for all colocalisation-positive and a sample of colocalisation-negative tests. Upon inspection, we decided either to merge peaks into one signal when they were considered too close to reliably tell which OpenTargets peak was driving the colocalisation result (retaining the one with the smallest p-value), or manually assigned the coloc test to one of the peaks in the region when this could be clearly distinguished. For regions containing only tests with no evidence of colocalisation and with more than one peak, we merged peaks < 30 kb apart. The 30kb threshold was chosen from examination of the distance between merged/unmerged peaks in visually inspected cases. When no merge happened, we let colocalisation-negative tests be assigned to multiple peaks, since coloc had rejected colocalisation with either peak. The final number of peaks was 1390, and the colocalisation tests assigned to them are summarised in S2 Table.

### Determinants of colocalisation

We used the top GWAS peak SNP as a proxy for the causal variant and ran a logistic regression to estimate the log(odds) of colocalisation in any test performed. For the analysis using the OTG colocalisation results the independent variables were the p-value of the GWAS lead SNP, the European MAF and the distance in kilo base-pairs from the lead SNP to the proximal transcription start site (TSS). For the in-house colocalisation analysis of IMDs, an indicator variable was included to capture whether the lead SNP overlapped a predicted active enhancer in immune cells [12].

### Colocalisation enrichment analysis in OTG

We tested whether cell types or tissues (37) were enriched for colocalisation for immune mediated diseases (3,032 coloc tests), endocrine system disease (8,136 coloc tests) and cardiovascular disease (29,265 coloc tests). For each disease group we ran a logistic regression with significant colocalisation as outcome and independent indicator variables for each cell type or tissue and the total number of tests per cell type or tissue. To account for the clustering structure with multiple observations per GWAS peak, cluster-robust standard errors were computed using the vcovCL() function from the *sandwich* package in R. The resulting p-values were Bonferroni-adjusted by the number of cell types or tissues tested. The reference tissue was “adipose”.

### Colocalisation enrichment analysis in IMDs

We grouped colocalisation tests by nominal cell types: B cells, CD4 T cells, CD8 T cells, DC, monocytes and natural killer or whole blood. We estimated the log(odds) of colocalisation using a logistic regression model with cell type as independent variable and a covariate for the number of colocalisation tests per cell type. We use whole blood as the reference category. To assess the enrichment of granularity and sample composition covariates, we excluded whole blood, and re-ran the logistic regression with cell type, sorted vs single cell, mixture of patients/control samples vs control, resting vs stimulated cells as independent variables. We use natural killer cells, sorted cells, controls and unstimulated cells as baseline categories. To account for the clustering structure with multiple observations per GWAS peak, cluster-robust standard errors were computed using the vcovCL() function from the *sandwich* package in R. Similar analysis was performed to estimate the log(odds) for colocalisation by nominal cell type for each IMD. Only IMDs with at least 10 coloc tests were considered.

### Co-expression of multi-coloc genes in the same cell type

For this analysis we excluded colocalisation tests in whole blood. We grouped coloc tests in the following cell types: B cells, CD4 T cells, CD8 T cells, dendritic cells, monocytes, neutrophils, natural killer cells, gamma-delta T cells, MAIT, double negative T cells and platelets. To avoid redundancy of coloc tests due to overlapping peaks from different IMDs testing the same eQTLs, we only keep one peak randomly, for those with the top fine-mapped SNP within 30KB. We chose 30KB to match our definition of independent peaks (see Definition of GWAS peaks section). This reduced the dataset to 549 peaks. For each gene-gene pair combination tested for colocalisation with the same peak, we annotated whether the two genes significantly colocalised and whether they were expressed in the same cell type. A logistic regression model was employed to estimate the odds ratio for co-localisation among genes expressed within the same cell type. To account for the clustering structure inherent to each peak, cluster-robust standard errors were computed using the vcovCL() function from the *sandwich* package in R.

### eQTL LD for multi-coloc genes in the same or different cell type

For gene pairs colocalising with the same peak using eQTL studies from European ancestry we calculated the squared correlation of the eQTL lead SNPs using a random sample of 40,000 individuals of European origin from UKBB.

### Effect of cross-mappability between gene pairs in the proportion of multi-coloc genes

For this analysis we used the pre-computed cross-mappability indices between genes to identify pairs of genes with potential mappability artifacts by Saha et. al. [18]. Cross-mappability from gene A to gene B, crossmap(A,B), is the number of gene A’s k-mers (75-mers from exons and 36-mers from UTRs) whose alignment start within exonic or untranslated regions of gene B. As cross-mappability between two genes is not necessarily symmetric, for each gene pair we selected the highest mappability index. For each peak we considered all pairs of colocalising genes and for each pair of genes that showed evidence of mappability issues we excluded one gene. Subsequently, we calculated the proportion of peaks with 1 or more colocalised genes per peak.

### Expression correlation of multi-coloc genes

For this analysis we selected peaks with two or more significant colocalisation genes in the ImmunextUT dataset which has available gene expression data, resulting in 53 peaks. For each gene-peak pair, we selected the coloc test (performed in different cell types) with the highest PP(H4), resulting in 1,175 coloc tests of which 132 were significant. This is to avoid dealing with multiple potentially redundant tests when a significant colocalising gene is detected in multiple studies. By selecting the eQTL study with the highest pH4, we ensure we have a good number of tests. Next, for each peak we tabled all pair-wise combinations of significant colocalising genes and calculated the gene expression (log + 1) Pearson-correlation squared matching the cell types of the coloc tests. As control, for each pair of significant genes, we randomly replaced one of the genes with a non-significant gene tested for the same peak in the same eQTL study as the gene to replace, and we calculated the expression correlation as before. Of note, non-significant genes used as controls were only selected once. This resulted in the same number of correlations for significant coloc tests and controls. A linear regression model with the outcome variable the squared expression correlation of gene pairs and independent variable colocalisation status (significant vs. non-significant) was fit. To account for the clustering structure inherent to each peak, cluster-robust standard errors were computed using the vcovCL() function from the *sandwich* package in R.

In addition, we evaluated whether pairs of co-colocalised genes tend to be more proximal than control pairs. We fit a linear model with outcome variable the distance between the transcription start site of the genes in base pairs and explanatory variable whether the pair of genes co-colocalised with the same peak or not. Here, we also computed cluster-robust standard errors using the vcovCL() function from the *sandwich* package in R to account for the multiple colocalisation tests per peak.

### Testing whether GWAS peak-lead SNPs tend to overlap regulatory elements for colocalising eGenes

We used publicly available maps for enhancer-gene and promoter-gene regulatory interactions using the scE2G multiome model for dendritic cells, monocytes, B cells, CD4 and CD8 T cells and natural killer cells from human peripheral blood mononuclear cells [12]. The input for the scE2G multiome model is paired scRNA and scATAC-seq data, and using a classifier based on logistic regression the model predicts which chromatin accessible elements act as enhancers to regulate which genes in a given cell population. Each enhancer-gene pair is annotated with a score (E2G score) corresponding to the probability of a regulatory effect. Active enhancer-gene pairs were defined as those with an E2G score > 0.164, as previously described [12]. Subsequently, we selected GWAS peaks for which the lead SNP overlapped a predicted active enhancer in any of the immune cell types tested (monocytes, dendritic cells, CD4 T, CD8 T, NK and B cells), under the assumption that the causal variant regulates the enhancer activity. We found that ~21% of the peaks overlapped an active enhancer. Then, for genes that had more than one coloc test with a peak within a nominal cell type (monocytes, dendritic cells, CD4 T, CD8 T, NK and B cells) we selected the coloc test with the maximum PP(H4). A mixed-effects logistic regression model was fitted using the glmer() function from the *lme4* package in R. The binary outcome variable indicated whether the enhancer overlapping the peak lead SNP interacted with the eGene promoter in the same cell type of the eQTL study. Fixed effects included (i) an indicator variable denoting whether the colocalisation (coloc) test was significant and (ii) the number of colocalisation significant genes associated with a peak. An interaction term between these two predictors was included to test for effect modification. To account for non-independence arising from multiple coloc tests per peak, a random intercept for peak was incorporated into the model.

## Supporting information

S1 Fig Summary of eQTL studies. Diversity of the study design of the 101 eQTL experiments used in our analysis (x-axis) from 12 studies (y-axis).
(TIF)

S2 Fig Colocalisation in IMDs and related diseases. A. Proportion of significant coloc tests (y-axis) as a function of the posterior probability of colocalisation (PP(H4)). B. Estimated false discovery rate (FDR) (y-axis) as a function of the posterior probability for colocalisation (PP(H4)) (x-axis). The vertical and horizontal lines indicate the threshold selected from downstream analysis that corresponds to 5% FDR. C. Proportion of peaks with evidence of colocalisation comparing IMDs (blue bar), diseases with underlying chronic inflammation but not considered immune mediated, OTH (purple bar) or psychiatric disorders, PSD (green bar). The error bars correspond to the 95% confidence interval. D. Number of gene TSS between an IMD GWAS lead SNP and its closest significant colocalising eGene (truncated at 8). The upper plot was done considering all annotated genes while the bottom one was restricted to protein coding genes.
(TIF)

S3 Fig Number of significant colocalisations by eQTL study in IMDs. For each of the eQTL studies (x-axis) the dots show the absolute number of significant coloc tests across all IMDs (y-axis). eQTLGen is shown in black.
(TIF)

S4 Fig Effect of cross-mappability issues on the distribution of multi-coloc genes per peak. For each pair of colocalising genes within each peak with evidence of mappability issues we excluded one of the genes. The plot shows the distribution of the proportion of colocalised genes per peak in the IMD (black bars) or OTG (grey bars) analysis.
(TIF)

S1 Table Description of the eQTL studies used for the colocalisation analysis with IMDs. (XLSX)

S2 Table Summary of QC for assignment of coloc tests to peaks by GWAS dataset. (XLSX)

S3 Table Definition of GWAS peaks used in the IMD colocalisation analysis. (TSV)

S4 Table Summary of colocalisation results for the IMD analysis. (TSV)
